# Factor VII promotes hepatocellular carcinoma progression through ERK-TSC signaling

**DOI:** 10.1038/cddiscovery.2015.51

**Published:** 2015-11-30

**Authors:** M-C Tsai, K-D Chen, C-C Wang, K-T Huang, C-H Wu, I-Y Kuo, L-Y Chen, T-H Hu, S Goto, T Nakano, A Dorling, J H McVey, C-L Chen, C-C Lin

**Affiliations:** 1 Division of Hepato-Gastroenterology, Department of Internal Medicine, Kaohsiung Chang Gung Memorial Hospital, Kaohsiung, Taiwan; 2 Graduate Institute of Clinical Medical Sciences, Chang Gung University College of Medicine, Kaohsiung, Taiwan; 3 Center for Translational Research in Biomedical Sciences, Liver Transplantation Program and Department of Surgery, Kaohsiung Chang Gung Memorial Hospital, Kaohsiung, Taiwan; 4 Fukuoka Institution of Occupational Health, Fukuoka, Japan; 5 Division of Transplantation Immunology and Mucosal Biology, Guy’s Hospital, King’s College London, MRC Centre for Transplantation, London, UK; 6 Department of Biochemical Sciences, Faculty of Health and Medical Sciences, University of Surrey, Guildford, UK

## Abstract

We previously demonstrated PAR2 starts upstreamed with tissue factor (TF) and factor VII (FVII), inhibited autophagy via mTOR signaling in HCC. However, the mechanism underlying for merging functions of PAR2 with the coagulation system in HCC progression remained unclear. The present study aimed to investigate the role of TF, FVII and PAR2 in tumor progression of HCC. The expressions of TF, FVII and PAR2 from HCC specimens were evaluated by immunohistochemical stains and western blotting. We found that the expression of FVII, but not TF and PAR2, directly related to the vascular invasion and the clinical staging. Importantly, a lower level of FVII expression was significantly associated with the longer disease-free survival. The addition of FVII but not TF induced the expression of PAR2 and phosphorylation of ERK1/2, whereas knockdown of FVII decreased PAR2 expression and ERK1/2 phosphorylation in HCC cell lines. Furthermore, levels of phosphor-TSC2 (Ser664) were increased after treatment with FVII and PAR2 agonist whereas these were significantly abolished in the presence of a potent and specific MEK/ERK inhibitor U0126. Moreover, mTOR knockdown highly reduced Hep3B migration, which could be reverted by FVII but not TF and PAR2. These results indicated that FVII/PAR2 signaling through MEK/ERK and TSC2 axis for mTOR activation has potent effects on the migration of HCC cells. In addition, FVII/PAR2 signaling elicits an mTOR-independent signaling, which promotes hepatoma cell migration in consistent with the clinical observations. Our study indicates that levels of FVII, but not TF, are associated with tumor migration and invasiveness in HCC, and provides clues that evaluation of FVII expression in HCC may be useful as a prognostic indicator in patients with HCC and may form an alternative target for further therapy.

## INTRODUCTION

Hepatocellular carcinoma (HCC) is the seventh most common malignancy worldwide.^1^ The current options for the treatment of this cancer consist of surgical resection, liver transplantation, percutaneous locoregional ablation therapy and chemotherapy including molecular targeted therapy.^2,3^ However, the high recurrence rate is still a major concern after any treatment, although the underlying mechanisms are still not fully defined.^4^ A better understanding of these mechanisms may lead to novel therapeutic approaches. Recent advances have highlighted that protease-activated receptor-2 (PAR2) has a regulatory function in HCC cell invasion.^5^ Therefore, a crucial role for a PAR2-mediated signaling pathway in HCC progression can be hypothesized.

Coagulation factor VII (FVII) participates in the initiation of the extrinsic pathway by binding to tissue factor (TF).^6^ Formation of TF-FVIIa complex leads to activation of coagulation cascade and platelet activation.^7^ In addition, increasing evidence indicates that the TF-FVII complex is also involved in physiological and pathophysiological processes involved in the development and spread of cancer, including angiogenesis, tumor migration and invasion and cell survival.^8–10^ On tumor cells, TF/FVII-dependent signaling primarily activates PAR2, which belongs to a family of four G-protein-coupled receptors,^11^ and thereby shapes the tumor microenvironment by inducing an array of pro-angiogenic and immune modulating cytokines, chemokines and growth factors.^12^ Several studies have documented that increased expression of TF mediated by TF-FVII-PAR2 signaling correlates with aggressive phenotypes in colorectal, breast, pancreatic cancers and gliomas.^13,14^ Hence, targeting the pathway may be an effective approach for cancer therapy. However, the role of TF-FVII-PAR2 signaling in HCC has not been well investigated.

Herein, we present evidence that FVII-PAR2 signaling but not TF plays an important role in HCC cell migration and invasion mediated through the p44/42 mitogen-activated protein kinase (MAPK) pathway. Of importance, our study indicates that FVII plays a critical role in HCC tumor biology regulating TF-FVII-PAR2 signaling.

## Results

### Correlation of TF, FVIIa and PAR2 with clinicopathologic characteristics of 100 HCC patients

**Figure 1:**
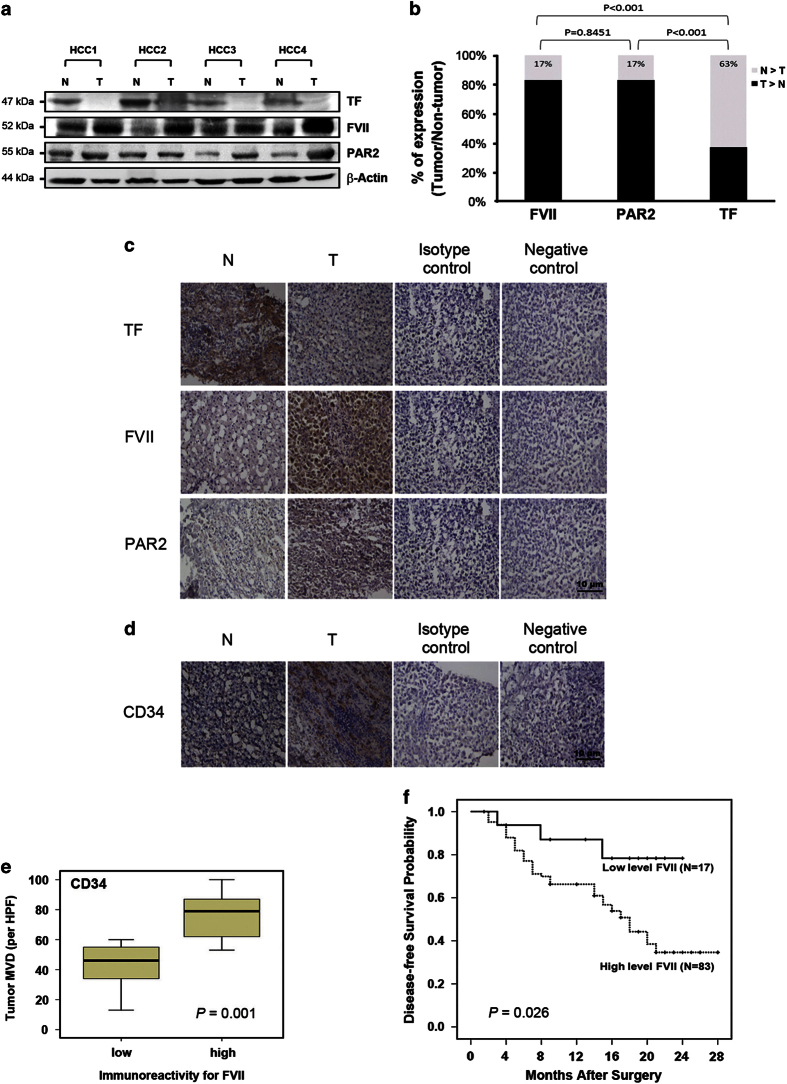
FVII overexpression correlates with PAR2 in human HCC and disease-free survival. (**a**) Western blot analysis of TF, FVII and PAR2 in four representative HCC tissues (T) and their paired non-tumor (N) tissues. *β*-Actin was used as a loading control. (**b**) FVII and PAR2 over expressed (defined as greater than onefold increase) in human HCC tumor compared with adjacent non-tumor tissues, and they are positively correlated with no significant difference among 100 cases (*P*=0.845). (**c**) The typical profiles of IHC staining with anti-TF, FVII or PAR2 antibody illustrated that greater immunoreactivity for FVII and PAR2 were found in the tumor region than in the non-tumor. (**d**) The endothelial cells of blood vessels were stained by IHC with an anti-CD34 antibody in one paired HCC and non-tumor tissues. (**e**) Relationship between FVII expression and microvessel count in paired HCC and non-tumor tissues. The lines through the idle of the boxes represent the median, while the top and bottom of the boxes are the 25th and 75th percentiles. The error bars represent measurement range, original magnification: ×200. (**f**) Overexpression of FVII (defined as greater than onefold increase in tumor tissue compared to its paired non-tumor tissue) is associated with the recurrence in patients with HCC after curative resection.

The expression of TF, FVII and PAR2 were examined by western blot analysis in 100 pairs of HCC patients (representative pairs shown in Figures 1a and b). Compared with the paired non-tumor tissues, high levels (defined as greater than onefold increase) of both FVII and PAR2 expression in 83 of 100 HCC cases. In contrast, the expression of TF was greater in only 37% of HCC specimens. Furthermore, an association analysis showed no significant difference between FVII and PAR2 expression among these 100 HCC specimens (*P*=0.845). We further examined the correlation between the expression of TF, FVII and PAR2 and clinicopathologic parameters (Table 1). The results indicated that TNM stage (*P*<0.001), tumor capsule (*P*=0.029) and microvenous invasion (*P*=0.003) were significantly correlated with FVII expression. But the size and number of tumors were not associated with FVII expression. However, microvenous invasion (*P*=0.059) was almost significantly correlated with PAR2 expression. The findings suggested that FVII and PAR2 may be involved in HCC progression.

### Correlation between FVII and microvascular density

To further investigate the correlations between FVII, TF and PAR2 and tumor characteristics, the levels of FVII, TF and PAR2 were compared by immunohistochemistry (IHC; Figure 1c). Consistently, compared with the non-tumor tissues, a profound increase in expression of FVII and PAR2 was detected in HCC tissue (85 and 80% of samples, respectively). Likewise, increased expression of TF was detected in 33.3% of samples. To estimate the correlation between microvascular density (MVD) and expression of FVII and PAR2, specific staining of capillary-like vessels by anti-CD34 was examined (Figure 1d). The MVD was significantly higher in tumors with high-level FVII than those with low level of FVII (median, 46 *versus* 81/high-power field (HPF), *P*=0.001; Figure 1e).

### FVII levels predict disease-free survival in HCC patients after curative resection

All 100 HCC patients undergoing hepatectomy were followed up at regular intervals until death or until the time of this writing, and the median duration of follow-up was 18 months (range 1–27 months). The tumors in these patients were categorized as high (*n*=83) or low (*n*=17) expressers of FVII according to the results of the western blots. Patients with high FVII levels had a significantly shorter disease-free survival than those with low FVII levels (*P*=0.026; Figure 1f). However, there was no correlation between tissue TF and PAR2 levels and disease-free survival rates (data not shown).

### FVII regulates p-ERK1/2 via PAR2 *in vitro*

**Figure 2:**
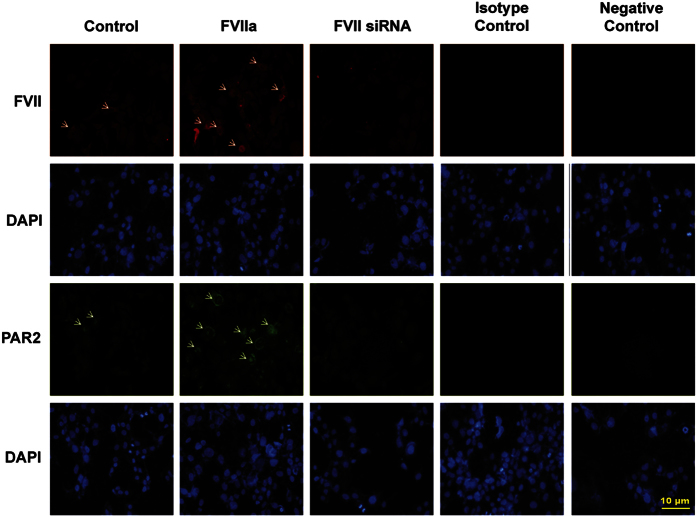
The effects of FVIIa on the expression of PAR2 in Hep3B cell line. Immunofluorescence was also used to detect the upregulation and downregulation of PAR2 in Hep3B cells treated with activated FVII (FVIIa) and FVII siRNA, respectively. In cells treated with FVIIa, prominent PAR2 staining was detected in the cell membrane (arrow, magnification ×200).

**Figure 3:**
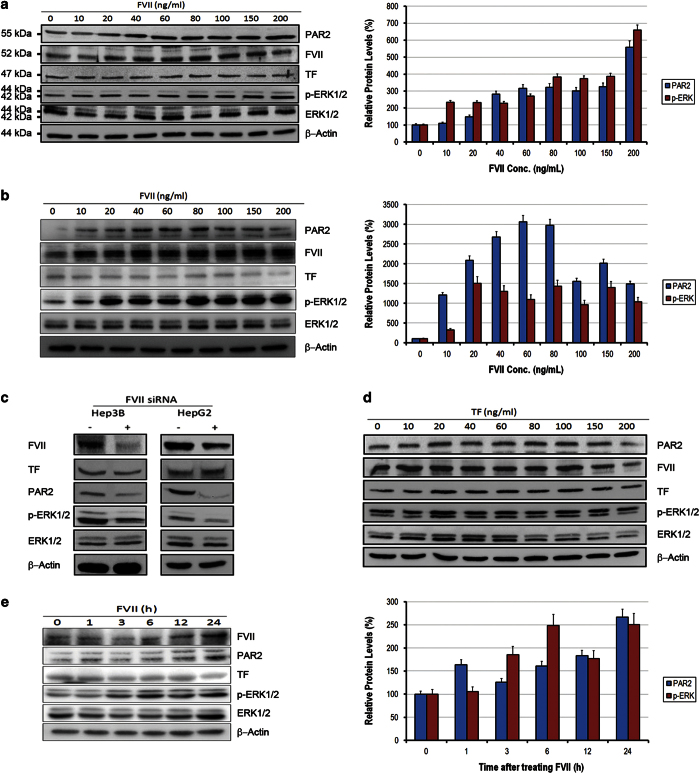
FVII, but not TF, increases PAR2 expression and p-ERK1/2 levels in dose-dependent and time-dependent manners. Quiescent monolayers of Hep3B (**a**) and HepG2 (**b**) cells (1×105) were treated with a control serum-free medium or medium supplemented with recombinant FVIIa (NovoSeven RT) for series of concentrations (10, 20, 40, 60, 80, 100, 150 and 200 ng/ml). After 6 h, cells were harvested and detected for TF, FVII, PAR2, p-ERK1/2 and *β*-actin by western blot analysis. The levels of PAR2 and p-ERK1/2 were gradually increased both in Hep3B and HepG2 cells. (**c**) The levels of PAR2 and p-ERK1/2 were significantly reduced by knockdown of FVII using siRNA both in Hep3B and HepG2 cells. (**d**) Hep3B cells were treated with a control serum-free medium or medium supplemented with TF (Merck) for series of concentrations (10, 20, 40, 60, 80, 100, 150 and 200 ng/ml). After 6 h, cells were harvested and detected for TF, FVII, PAR2, p-ERK1/2 and *β*-actin by western blot analysis. The levels of PAR2 and p-ERK1/2 were not gradually increased by increasing concentration of TF. (**e**) Hep3B cells were treated with medium supplemented with FVIIa for a time-series analysis (0, 1, 3, 6, 12 and 24 h). The levels of PAR2, p-ERK1/2 but not TF were gradually increased in a time-dependent manner.

We primarily examined four HCC cell lines (Hep3B, HepG2, Huh and PLC) and two breast cancer cell lines as positive control (MDA-MB-231 and MCF7)^15,16^ to assess whether these cells endogenously express TF, FVII and PAR2. As expected, western blot analysis showed that HCC cell lines can express TF, FVII and PAR2 proteins (Supplementary Figure 1). To determine how TF/FVII/PAR2 signaling is working in HCC cells, activated FVII (FVIIa, 200 ng/ml) were added to the cultures of Hep3B cell line, and the expression of PAR2 was qualitatively observed on immunofluorescence (IF) microscopy. The levels of PAR2 and phosphorylated ERK1/2 (p-ERK1/2) were quantitatively determined by western blot analysis. As shown in Figure 2, FVIIa increased the expression of PAR2 as detected qualitatively by IF staining, which shown marked expression of PAR2 in cell membrane after FVIIa treatment. The dose-dependent increase of PAR2 and p-ERK1/2 were observed in both Hep3B and HepG2 cell lines by western blot (Figures 3a and b), whereas knockdown of FVII by siRNA demonstrated notable reduction in the levels of PAR2 and p-ERK1/2 (Figure 3c). However, TF treatment (200 ng/ml) did not alter PAR2 and p-ERK1/2 levels (Figure 3d). Furthermore, FVIIa also exhibited a time-dependent upregulation in levels of PAR2 and p-ERK1/2 in Hep3B cells (Figure 3e).

### FVII/PAR2 signaling regulates TSC2/mTOR phosphorylation via ERK

The mammalian target of rapamycin (mTOR) is frequently dysregulated in various cancer cells and abnormally activated in a proportion of HCC patients,^17^ which is under investigation as a potential target to suppress liver tumor growth and metastasis.^18,19^ We have previously demonstrated that TF/FVII/PAR2 signaling negatively regulates autophagy via mTOR activation, which promotes proliferation of Hep3B cells *in vitro*.^20^ To investigate the potential role of ERK1/2 in mTOR activation by this coagulation signaling, we first examined the effect of a specific MEK/ERK inhibitor U0126 in FVII/PAR2-mediated mTOR activation.

**Figure 4:**
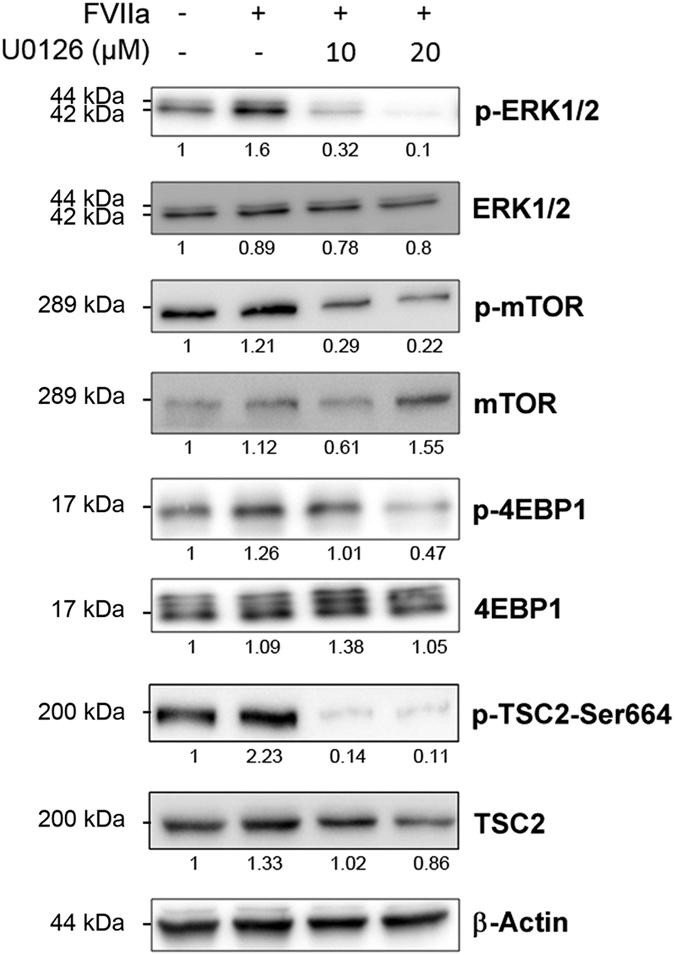
The effects of MEK/ERK inhibition by U0126 on the levels of p-ERK, p-mTOR, p-4EBP1 and p-TSC2-Ser664 in Hep3B cells. Hep3B cells were treated with FVIIa (200 ng/ml) in the absence or presence of U0126 (10 or 20 *μ*M) 24 h, and total amount of cells were harvested and detected for ERK1/2, p-ERK1/2, mTOR, p-mTOR, 4EBP1, p-4EBP1, p-TSC2-Ser664, TSC2 and *β*-actin by western blot assay. All results are expressed as the mean±S.D. from three independent experiments. The levels of p-ERK1/2, p-mTOR, p-4EBP1 as well as p-TSC2-Ser664 induced by FVIIa were significantly abolished by U0126.

Western blot analysis demonstrated that levels of the phosphorylated ERK1/2 (p-ERK), mTOR (p-mTOR, Ser^2448^, active form) as well as the downstream substrate 4EBP1 (p-4EBP1, Ser^65^/Thr^70^), which were activated by FVII/PAR2 signaling were suppressed by U0126 treatment in a dose-dependent manner (Figure 4). In addition, levels of the phosphorylated TSC2 (p-Ser^664^) induced by FVII/PAR2 signaling were also reverted by U0126 treatment indicated that mTOR activation by FVII/PAR2 activities relies upon ERK-mediated TSC2 phosphorylation.

### Knockdown of FVII and PAR2 inhibits cell invasion and migration via MEK/ERK and TSC2/mTOR pathway

**Figure 5:**
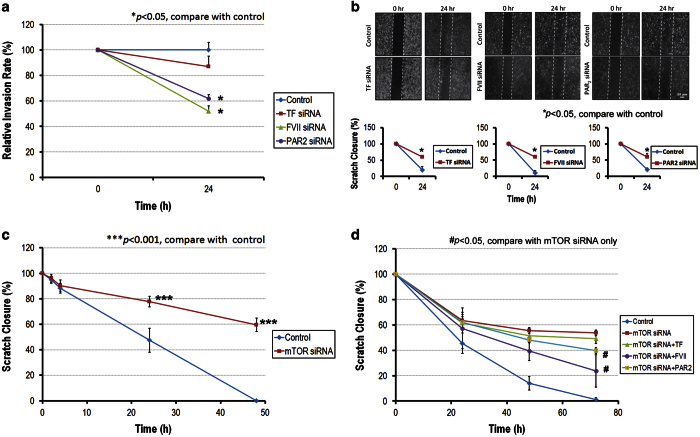
Effects of TF/FVII/PAR2 on the tumor phenotype. (**a**) Invasion: Hep3B cells transfected with TF siRNA (30 nM), FVII siRNA (30 nM) or PAR2 siRNA (30 nM) for 48 h were placed on top of a Matrigel barrier. At the end of a 24-h incubation period at 37 °C, the number of cells that migrated across the Matrigel barrier to the underside of the membrane was determined. (**b**) Migration: Hep3B cells transfected with TF siRNA (30 nM), FVII siRNA (30 nM) or PAR2 siRNA (30 nM) for 48 h were cultivated to optical confluence, and a scratch was performed subsequently on a medium. After 24 h of incubation at 37 °C, cell migration was measured by counting as described in experimental procedures. (**c**) Hep3B cells cultured in 1% serum were pre-transfected with mTOR siRNA (30 nM) for 24 h. Knockdown of mTOR significantly attenuated the ability of Hep3B to close the scratch. (**d**) The attenuated migration of Hep3B cells by mTOR knockdown was reverted partly by FVIIa and PAR2 agonist, but not TF treatment. Representative results from three independent experiments are shown.

**Figure 6:**
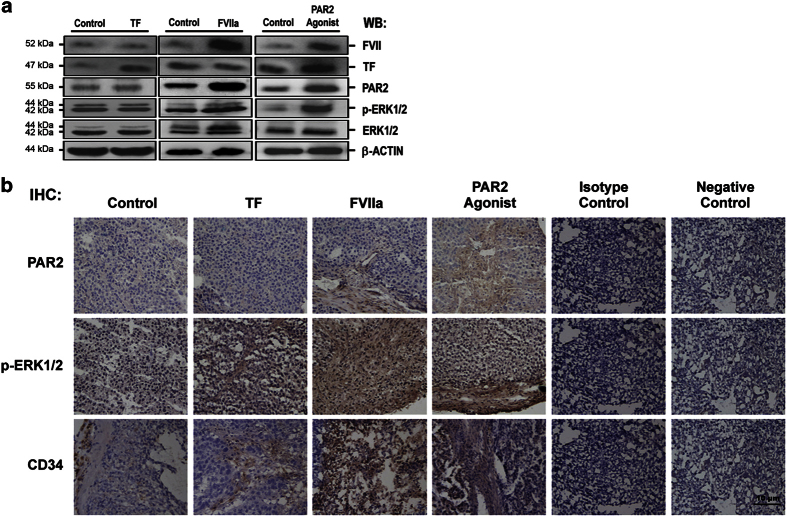
The effect of FVII on PAR2, p-ERK1/2 and CD34 levels in mouse xenograft model. Subcutaneously grown HepG2 tumors were injected into NOD/SCID mice. TF, FVIIa or PAR2 agonist was directly injected into the grafted tumors. (**a**) The expression levels of the three coagulation factors and p-ERK1/2 in tumor tissue were determined by western blot analyses. (**b**) Immunohistochemistry for PAR2, p-ERK1/2 and CD34 in tumor tissue treated with TF, FVIIa and PAR2 were also determined. Representative results from five independent animal experiments are shown. FVII significantly increased levels of PAR2, p-ERK1/2 and the microvessel density (MVD) in HepG2 xenograft.

We further investigated the role of TF/FVII/PAR2 signaling in tumor cell invasion and migration. As shown in Figure 6, the invasion of Hep3B cells were significantly suppressed by knockdown of FVII and PAR2 genes, however, no significant effect of TF knockdown on Hep3B cell invasion was observed (Figure 5a). In the scratch migration assay, knockdown of TF, FVII and PAR2 significantly reduced migration of Hep3B cells (Figure 5b). Furthermore, migration of Hep3B cells was significantly attenuated by mTOR knockdown (Figure 5c). Similar results were also observed in the invasion assay of Hep3B cells (data not shown). The results indicated that the signal through FVII/PAR2/mTOR axis via ERK activity was involved in HCC cell migration and invasion. Moreover, Hep3B cells reveal reduced migration activity upon knockdown of mTOR was significantly restored by treatment of FVIIa and PAR2 agonist (Figure 5d). This result suggested that FVII/PAR2 signaling might promote HCC cell migration through both mTOR-dependent and -independent pathways.

### FVII increased expression of p-ERK1/2 and MVD in a xenograft mouse model

In a mouse xenograft tumor model, normal saline (control), FVIIa, TF and PAR2 agonist were injected into growing subcutaneous tumors every other day for 30 days. Although the number and size of tumors were similar (data not shown), injection of FVIIa, but not TF, increased the expression of PAR2 and levels of p-ERK1/2 in tumor cells, analyzed by both western blot and IHC (Figures 6a and b). Importantly, injection of FVIIa also increased MVD as evidenced by CD34 staining (Figure 6b).

## Discussion

Recent studies showed that PAR2 plays an important role in promoting HCC cell invasion, through the pathway of p42/p44 MAPKs.^5^ The upstream stimulators of TF and FVII, which form a binary complex, have also been shown to be involved with tumor biology in various cancers such as breast, colorectal cancer, as well as glioma.^14,15,21^ However, the role of TF and FVII in tumor progression of HCC has until now remained elusive.

It is universally accepted that FVII is manufactured by liver cells and circulates in the bloodstream, primarily in a zymogen (inactive) form, that is, FVII.^6^ Only ~1% of total FVII antigen circulates in the activated enzyme (FVIIa) form, which is insufficient to initiate coagulation under physiological conditions.^22^ The presence of procoagulant TF increases the conversion of inactive FVII, to the activated two-chain form, and this initiates the coagulation serine protease cascade when FVIIa forms a binary complex with the extracellular domain of TF. In addition to this central role in initiation of coagulation, recent studies have shown that ectopic synthesis of FVII by cancer cells activates cancer cell migration and metastasis.^16,23^ Recent studies further indicated that reduction of TF and FVIIa exerted an inhibitory effect on tumor growth in xenograft models of breast and colorectal cancer.^24,25^ Our previous study also demonstrated that TF/FVII/PAR2 signaling regulates autophagy mainly via mTOR signaling and impacts on cell survival of hepatoma cells.^20^ These findings suggest that FVII is indispensable in coagulation-mediated enhancement of tumor growth.

In the present study, despite a wide variation in the expression of TF, FVII and PAR2 by HCC tumors and non-tumor tissues, we observed a significant correlation between the expression of FVII and PAR2 by tumor specimens and a significant association between FVII and the clinical staging. Furthermore, patients with high levels of FVII expression in HCCs had a significantly worse disease-free survival than those tumors with low levels of FVII expression. Importantly, expression of FVII was exclusively associated with the presence of PAR2 but not downstream products of coagulation function such thrombin and its signal transduction effecter PAR1 (data not shown). Therefore, the clinical observations suggest that FVII plays an important role in tumorigenesis of HCC through a PAR2 signaling pathway.

Our *in vitro* data confirmed that FVII, but not soluble TF, upregulates the p-ERK1/2 mediated with PAR2. Moreover, the invasion- and migration-associated phenotypes could be effectively abolished by silencing FVII expression in HCC cells. Although many studies have revealed that TF-FVII-PAR2 signaling can initiate cell signal transduction in the pathogenesis of cancers and promotes cell migration and invasion,^14,15,21^ the detailed signaling transduction mechanisms responsible for the TF-FVII-PAR2 in HCC are not fully understood. Here, we showed that FVII and PAR2 agonist increase the phosphorylation of ERK1/2, however, no significant change in ERK phosphorylation was observed in TF treatment. We speculate that TF is expressed in HCC tissue with an excess amount, whereas the amount of FVII determines the proportion of TF that is engaged with FVII in the binary complex (active form) to regulate HCC tumor progression. Our data from the mouse xenograft model showed that injection of FVIIa increased vascular density but not the size and number of the tumors. The results are consistent with our clinical findings, which demonstrated that the expression of FVII by HCC was associated with vascular invasion and capsulations of tumor but not the number and size.

Recent studies have documented that the levels of TF expression in primary colorectal, breast and lung cancer correlate with aggressive cancer phenotypes and metastatic disease.^25–29^ Poon *et al.*^30^ indicated a correlation between TF expression and invasiveness in human HCC. However, in the present study, there was no correlation between TF and the presence of FVII, PAR2 or clinicopathological features. Our results are thus more consistent with the findings of Rullier *et al.*,^31^ who reported no correlation between TF and HCC progression.^31^ Similarly, other studies indicate that TF is not required for tumor growth.^32–34^ Taking all together, TF could play an important role in tumor progression in many but not all cancers. Although our previous findings have shown that TF indeed regulates survival of HCC cells via antagonizing autophagy through mTOR signaling, our results indicated that TF will not become a reliable prognostic marker at least in part for HCC progression.

It has been generally accepted that tumor cell motility is necessary for cancer dissemination.^35^ The molecular basis to acquire ability to colonize other organs by invading tumor cells has been long studied, but it still remains a largely unmet challenge in therapeutic control on metastatic dissemination.^36,37^ Especially in China and other East Asian countries, survival of HCC patients has improved due to advances in surgical techniques such as orthotopic liver transplantation and perioperative nursing care, long-term survival after surgical resection remains low owing to risk of recurrence and metastasis.^38,39^ Thus, to investigate the molecular mechanisms of HCC metastasis, it is of great interest to identify impaired metastatic suppressors responsible for the metastatic potential.

In this study, we have shown that knockdown of FVII and PAR2 significantly reduced HCC invasion and migration. We also showed that FVII-PAR2 signaling is a contributor to tumor migration in HCC, which may through both mTOR-dependent and mTOR-independent pathways. Inhibition of FVII-PAR2 signaling may thus represent an effective approach to targeted cancer therapy. Although there is an increased risk of bleeding with specific FVIIa inhibitors,^24,40^ a recent phase I study indicated that PCI-27483, a selective inhibitor of FVIIa, which promotes a 2.5- to 3.0-fold change in prothrombin time in animal studies, is nevertheless well tolerated in advanced pancreatic cancer patients.^41^ Although phase II study of this agent is ongoing, based on our results, it might prove to be an agent with therapeutic utility in HCC. Furthermore, we confirmed that metastatic suppressors NME1 and BHLHE41 were highly induced in Hep3B cells with FVII and PAR2 knockdown. Indeed, we also found treatment of FVII and PAR2 agonist significantly decrease expression of NME1 and BHLHE41 (Supplementary Figure 2). Moreover, another abundantly expressed member of NME gene family NME2 was highly increased only in HCC cells with FVII knockdown, but not in cells with PAR2 silencing (data not shown). These results indicate that metastatic suppressors could play a pivotal role in FVII-associated vascular invasion and poor prognosis in HCC patients. However, the details of the underlying mechanism need to be further clarified.

Taken together, we suggest that FVII levels in HCC patients may have prognostic significance, which may be particularly useful in the management of patients after HCC resection. It might be useful to offer adjuvant therapy after HCC resection to those patients with high levels of FVII expression, who have a poor disease-free survival rate. Further clinical studies are required to verify the prognostic efficacy of FVII levels in a large cohort of HCC patients, and the trials for adjuvant treatment after resection is approaching.

In summary, we have presented for the first time that increased FVII expression by tumor cells correlates with progression of HCC and acts as a poor prognostic factor after surgery and demonstrated that FVII/PAR2 through p-ERK1/2 signaling is involved in HCC progression. Importantly, we have presented evidence that FVII plays a novel role of the FVII/PAR2 signaling pathway in HCC, and provide mechanistic insights not only affecting mTOR but also modulating metastatic suppressors into our clinical observations. In the future, this work indicates that FVII may be a candidate marker for the development of prognostic and therapeutic strategies for HCC malignancy.

## Materials and Methods

### Patients and tissue samples

From August 2009 to August 2010, 100 patients who underwent curative hepatic resection for HCC at the Chang Gung Memorial Hospital, were recruited into this study. The diagnosis of HCC was based on the criteria of practice guidelines.^2,42^ All patients were followed in the outpatient clinic with regular surveillance for the recurrence by serum *α*-fetoprotein level and ultrasound every 3 months and/or contrast-enhanced computerized tomography scan if recurrent tumor was suspected. All patients were followed up until death or June 2012. The demographics, clinical character, pathological findings of HCC, recurrence and survival were recorded. The clinicopathologic characteristics of 100 HCC patients are summarized in Table 2. The study protocol was approved by the ethic committee of Chang Gung Memorial Hospital. Written informed consent was obtained from each patient. Tumor and adjacent tumor-free specimens (control tissues) were obtained immediately after surgical resection. The investigators who performed the laboratory studies of TF, FVIIa and PAR2 expression were blinded to the clinicopathologic data.

### IHC staining

The paraffin-embedded tissue blocks were sectioned for IHC. The slides were incubated overnight at 4 °C in humidified chambers with primary rabbit polyclonal anti-TF (Santa Cruz Biotechnology, Santa Cruz, CA, USA), PAR2 (Santa Cruz Biotechnology), FVII (Abcam, Cambridge, UK ) and CD34 (Santa Cruz Biotechnology) antibody. Antigen–antibody complexes were detected by the avidin–biotin–peroxidase method, using diaminobenzidine as a chromogenic substrate (DAKO, Carpinteria, CA, USA). Finally, the slides were counterstained with hematoxylin, and then examined under light microscopy.

### Western blot analysis

Total protein were homogenized with loading buffer, separated by 10% SDS-PAGE and transferred to nitrocellulose membranes. The membranes were probed with primary antibodies at 4 °C overnight, and then were incubated for 1 h with respective conjugated secondary antibodies (1 : 2000, Cell Signaling Technology, Billerica, MA, USA). Immunoreactive proteins were visualized by ECL western blot detection reagents (Millipore, Billerica, MA, USA), and quantitated using a G:BOX iChemi XL imaging systems (J&H Technology Co. Ltd., Bradenton, FL, USA). The western blot reactivity of TF, FVII and PAR2 were classified as high if staining in the tumor was higher than the non-tumor part of the biopsy.

### Immunofluorescence studies of human HCC tissue

Cells were fixed in 4% paraformaldehyde and incubated with primary antibody at 4 °C overnight and Texas Red (ThermoFisher, Waltham, MA, USA) or FITC-conjugated secondary antibody for 2 h at room temperature. Extensive washing with PBS was performed between each step and before mounting and examination by fluorescence microscopy (Olympus, Tokyo, Japan).

### Evaluation of MVD

MVD was determined by the presence of CD34 as descried by Weidner *et al*.^43^ Briefly, tumor and non-tumor tissue sections were scanned at low-power fields (×40) to find the areas that showed the most intense neovascularization (hot spots). Individual microvessels were counted in five fields at high power (×200). Any positively stained endothelial cell or endothelial-cell cluster that was clearly separated from adjacent microvessels, tumor cells and connective elements was considered to be a single and countable microvessel. Vessel lumens were not necessary for a structure to be defined a vessel lumen. The final MVD was the mean value obtained from the counts of five fields, which was expressed as the absolute number of microvessels per HPF.

### Cells and culture condition

Human hepatoma cell lines, Hep3B, HepG2 and PLC, and breast cancer cell lines, MDA-MB-231 and MCF7, obtained from American Type Culture Collection (Manassas, VA, USA), were cultivated in an incubator under a 5% carbon dioxide atmosphere at 37 °C in a relative humidity of 95% and were maintained in DMEM (Invitrogen, Karlsruhe, Germany) with 10% FBS (Invitrogen), 100 U/ml penicillin (Invitrogen), 100 mg/ml streptomycin (Invitrogen) and 4 mM L-glutamine (Invitrogen). Recombinant human TF and FVII were purchased from R&D Systems (Minneapolis, MN, USA). PAR1 agonist peptide TFLLR-NH2 and PAR2 agonist peptide SLIGKV-NH2 were purchased from Peptides International (Louisville, KY, USA).The working concentration of recombinant proteins and agonist peptides were 200 ng/ml.

### Tumor xenograft mouse model

Severe combined immunodeficiency (SCID) mice (4 weeks old) were housed under standard conditions and cared as per the institutional guidelines for animal care. For the xenograft tumor growth assay, HepG2 cells (5×10^6^) were injected subcutaneously into the right dorsal flank. Treatment, which was initiated when the tumor reached 5 mm in diameter, was through directly subcutaneous injection of FVIIa, TF or PAR2 (2 *μ*g/ml) agonist every other day. After 30 days, the mice were killed and the tumors were excised and extract protein for western blot analysis. The mice experiments were performed in accordance with U.S. National Institutes of Health guidelines, and the Chang Gung Institutional Animal Care and Use Committee Guide for Care and Use of Laboratory Animals. This study was conducted under the approval of Chang Gung Institutionally Animal Care and Use Committee (IACUC Approval NO 2011092001).

### siRNA transient transfection

The siRNA for knocking down target gene expression was obtained from Santa Cruz Biotechnology. In brief, cells were cultured in six-well plates and transfected with 30 nM target gene-specific siRNA or control siRNA using GenMute siRNA transfection reagent (signaGen Laboratories, Gaithersburg, MD, USA), and harvested for further analysis 48 h after transfection.

### Invasion assay

A polycarbonate filter (8 *μ*m pore size) precoated with Matrigel (Becton Dickinson, Franklin Lakes, NJ, USA) was used. Briefly, cells were resuspended in serum-free DMEM and seeded in 6-*μ*m PET transparent plates (Millipore). Prior to addition of the suspended cells, the Matrigel chambers (Becton Dickinson) were rehydrated at 37 °C in a humidified tissue culture incubator with DMEM. Approximately, 10% FCS was used as an invasion stimulus and added to the wells of the companion plate. After 24 h, invaded cells were fixed in 100% methanol for 2 min and subsequently stained in 0.1% crystal violet (Sigma-Aldrich, St. Louis, MO, USA) in ddH_2_O for 2 min. Invaded cells were counted under a light microscope counting 10 HPFs per chamber.

### Migration assay

Cells were seeded and grown to confluence in DMEM medium. A scratch was made using a Ibidi culture insert (Martinsried, Germany) and another medium change was performed. Wells were photographed using an inverted light microscope (Nikon, Dusseldorf, Germany) and cultivated for 24 h. After incubation the wells were photographed again. A grid system was used and the same coordinates were used for the photographs before and after incubation to compare the same spots. For quantitative analysis, the first and second photographs were correlated, and the number of cells migrated into the scratch were counted.

### Statistical analysis

Continuous data were presented as median and range, and compared between groups using the Mann-Whitney *U*-test. Categorical variables were compared using the chi-square test (or Fisher’s exact test where appropriate). Correlations between continuous variables were determined using the Spearman's rank correlation test. Survival rates were calculated using the Kaplan-Meier method, and the difference in survival was compared with the log-rank test. Receiver operating characteristic (ROC) curves were generated to capture the best trade-off between sensitivity and specificity of age, AFP level and tumor size for correlation with TF, FVII and PAR2. Statistical analysis was performed with the SPSS software package for Windows (SPSS 15.0 for Windows; SPSS Inc., Chicago, IL, USA). All *P*-values were derived from two-tailed tests and a level of <0.05 was accepted as statistically significant.

## Figures and Tables

**Table 1:** Correlation of TF, FVII and PAR2 expression with clinicopathological characteristics in 100 patients with HCC

*Variable*	*Total*	*FVII*		*P-value* a	*TF*		*P-value* a	*PAR2*		*P-value* a
		*T>N*	*N>T*		*T>N*	*N>T*		*T>N*	*N>T*	
*Age*				0.779			0.532			0.709
≥50 years	79	66	13		28	51		65	14	
<50 years	21	17	4		9	12		18	3	
*Sex*				0.270			0.257			0.779
Male	79	68	11		27	52		66	13	
Female	21	15	7		10	11		17	4	
*Cirrhosis*				0.661			0.993			0.132
Present	54	44	10		20	34		42	12	
Absent	46	39	7		17	29		41	5	
*Hepatitis*				0.707			0.454			0.321
B	57	48	9		21	36		48	9	
C	22	17	5		9	13		18	4	
B+C	4	4	0		0	4		2	2	
NBNC	17	14	3		7	10		15	2	
*AFP*				0.270			0.619			0.222
≥200 ng/ml	30	23	7		10	20		27	3	
<200 ng/ml	70	60	10		27	43		56	14	
*Tumor size*				0.316			0.847			0.940
≥5 cm	42	33	9		16	26		35	7	
<5 cm	58	50	8		21	37		48	10	
*TNM stage*				<0.001			0.880			0.187
I	24	13	11		8	16		17	7	
II	51	50	1		20	31		44	7	
III	25	20	5		9	16		22	3	
*Encapsulation*				0.029			0.199			0.703
Present	21	14	7		5	16		17	4	
Absent	77	68	9		30	47		65	12	
*Microvenous invasion*				0.003			0.593			0.059
Present	56	52	4		22	34		50	6	
Absent	44	31	13		15	29		33	11	
*Satellite nodule*				0.195			0.304			0.566
Present	24	22	2		11	13		19	5	
Absent	79	61	15		26	50		64	12	

Abbreviations: N, non-tumor; T, tumor.

a Categorical data were compared using a chi-square test.

**Table 2:** Clinicopathological features of 100 patients with HCC undergoing hepatectomy

*Patient demographics*	
Age (years; median (range))	59 (34–83)
Sex (M : F)	79 : 21
AFP (ng/ml; median (range))	22 (2–342193)
Tumor size (cm; median (range))^a^	3.9 (1.0–19.3)
Liver cirrhosis (+; %)	54 (54)
Hepatitis (B : C : B+C : NBNC)	57 : 22 : 4 : 17
TNM stage (I : II : III)	24 : 51 : 25
	
*Pathological features*	
Capsule (yes : no)	21 : 77
Satellite nodule (yes : no)	24 : 76
Microvascular invasion (yes : no)	56 : 44
Histological grade (I : II : III)	9 : 82 : 5

a Measured by the length of the largest tumor nodule.

## Abbreviations

HCC: Hepatocellular carcinoma
FVII: Factor VII
TF: tissue factor
PAR2: Protease-activated receptor-2
ERK: extracellular-signal-regulated kinase
TSC: tuberous sclerosis protein.

## References

[bib1] Omata M , Lesmana LA , Tateishi R , Chen PJ , Lin SM , Yoshida H et al. Asian Pacific Association for the Study of the Liver consensus recommendations on hepatocellular carcinoma. Hepatol Int 2010; 4: 439–474. 20827404 10.1007/s12072-010-9165-7 PMC2900561

[bib2] Bruix J , Sherman M . Management of hepatocellular carcinoma. Hepatology 2005; 42: 1208–1236. 16250051 10.1002/hep.20933

[bib3] Shen YC , Hsu C , Cheng AL . Molecular targeted therapy for advanced hepatocellular carcinoma: current status and future perspectives. J Gastroenterol 2010; 45: 794–807. 20567987 10.1007/s00535-010-0270-0

[bib4] Avila MA , Berasain C , Sangro B , Prieto J . New therapies for hepatocellular carcinoma. Oncogene 2006; 25: 3866–3884. 16799628 10.1038/sj.onc.1209550

[bib5] Kaufmann R , Oettel C , Horn A , Halbhuber KJ , Eitner A , Krieg R et al. Met receptor tyrosine kinase transactivation is involved in proteinase-activated receptor-2-mediated hepatocellular carcinoma cell invasion. Carcinogenesis 2009; 30: 1487–1496. 19546160 10.1093/carcin/bgp153

[bib6] Furie B , Furie BC . The molecular basis of blood coagulation. Cell 1988; 53: 505–518. 3286010 10.1016/0092-8674(88)90567-3

[bib7] Nemerson Y . Tissue factor and hemostasis. Blood 1988; 71: 1–8. 3275472

[bib8] Abe K , Shoji M , Chen J , Bierhaus A , Danave I , Micko C et al. Regulation of vascular endothelial growth factor production and angiogenesis by the cytoplasmic tail of tissue factor. Proc Natl Acad Sci USA 1999; 96: 8663–8668. 10411932 10.1073/pnas.96.15.8663 PMC17573

[bib9] Mueller BM , Ruf W . Requirement for binding of catalytically active factor VIIa in tissue factor-dependent experimental metastasis. J Clin Invest 1998; 101: 1372–1378. 9525979 10.1172/JCI930 PMC508714

[bib10] Versteeg HH , Spek CA , Richel DJ , Peppelenbosch MP . Coagulation factors VIIa and Xa inhibit apoptosis and anoikis. Oncogene 2004; 23: 410–417. 14724569 10.1038/sj.onc.1207066

[bib11] Camerer E , Huang W , Coughlin SR . Tissue factor- and factor X-dependent activation of protease-activated receptor 2 by factor VIIa. Proc Natl Acad Sci USA 2000; 97: 5255–5260. 10805786 10.1073/pnas.97.10.5255 PMC25815

[bib12] Schaffner F , Ruf W . Tissue factor and PAR2 signaling in the tumor microenvironment. Arterioscler Thromb Vasc Biol 2009; 29: 1999–2004. 19661489 10.1161/ATVBAHA.108.177428 PMC2806842

[bib13] Milsom C , Anderson GM , Weitz JI , Rak J . Elevated tissue factor procoagulant activity in CD133-positive cancer cells. J Thromb Haemost 2007; 5: 2550–2552. 17883595 10.1111/j.1538-7836.2007.02766.x

[bib14] Gessler F , Voss V , Dutzmann S , Seifert V , Gerlach R , Kogel D . Inhibition of tissue factor/protease-activated receptor-2 signaling limits proliferation, migration and invasion of malignant glioma cells. Neuroscience 2010; 165: 1312–1322. 19958818 10.1016/j.neuroscience.2009.11.049

[bib15] Hjortoe GM , Petersen LC , Albrektsen T , Sorensen BB , Norby PL , Mandal SK et al. Tissue factor-factor VIIa-specific up-regulation of IL-8 expression in MDA-MB-231 cells is mediated by PAR-2 and results in increased cell migration. Blood 2004; 103: 3029–3037. 15070680 10.1182/blood-2003-10-3417 PMC2837482

[bib16] Koizume S , Jin MS , Miyagi E , Hirahara F , Nakamura Y , Piao JH et al. Activation of cancer cell migration and invasion by ectopic synthesis of coagulation factor VII. Cancer Res 2006; 66: 9453–9460. 17018600 10.1158/0008-5472.CAN-06-1803

[bib17] Guertin DA , Sabatini DM . Defining the role of mTOR in cancer. Cancer Cell 2007; 12: 9–22. 17613433 10.1016/j.ccr.2007.05.008

[bib18] Wang Z , Zhou J , Fan J , Tan CJ , Qiu SJ , Yu Y et al. Sirolimus inhibits the growth and metastatic progression of hepatocellular carcinoma. J Cancer Res Clin Oncol 2009; 135: 715–722. 19002496 10.1007/s00432-008-0506-z PMC12160166

[bib19] Wang Z , Zhou J , Fan J , Qiu SJ , Yu Y , Huang XW et al. Effect of rapamycin alone and in combination with sorafenib in an orthotopic model of human hepatocellular carcinoma. Clin Cancer Res 2008; 14: 5124–5130. 18698030 10.1158/1078-0432.CCR-07-4774

[bib20] Chen KD , Wang CC , Tsai MC , Wu CH , Yang HJ , Chen LY et al. Interconnections between autophagy and the coagulation cascade in hepatocellular carcinoma. Cell Death Dis 2014; 5: e1244. 24853422 10.1038/cddis.2014.212 PMC4047908

[bib21] Guo D , Zhou H , Wu Y , Zhou F , Xu G , Wen H et al. Involvement of ERK1/2/NF-kappaB signal transduction pathway in TF/FVIIa/PAR2-induced proliferation and migration of colon cancer cell SW620. Tumour Biol 2011; 32: 921–930. 21625939 10.1007/s13277-011-0194-1

[bib22] Wildgoose P , Nemerson Y , Hansen LL , Nielsen FE , Glazer S , Hedner U . Measurement of basal levels of factor VIIa in hemophilia A and B patients. Blood 1992; 80: 25–28. 1611090

[bib23] Tang JQ , Fan Q , Wu WH , Jia ZC , Li H , Yang YM et al. Extrahepatic synthesis of coagulation factor VII by colorectal cancer cells promotes tumor invasion and metastasis. Chin Med J 2010; 123: 3559–3565. 22166631

[bib24] Liu Y , Jiang P , Capkova K , Xue D , Ye L , Sinha SC et al. Tissue factor-activated coagulation cascade in the tumor microenvironment is critical for tumor progression and an effective target for therapy. Cancer Res 2011; 71: 6492–6502. 21880589 10.1158/0008-5472.CAN-11-1145

[bib25] Yu JL , May L , Lhotak V , Shahrzad S , Shirasawa S , Weitz JI et al. Oncogenic events regulate tissue factor expression in colorectal cancer cells: implications for tumor progression and angiogenesis. Blood 2005; 105: 1734–1741. 15494427 10.1182/blood-2004-05-2042

[bib26] Seto S , Onodera H , Kaido T , Yoshikawa A , Ishigami S , Arii S et al. Tissue factor expression in human colorectal carcinoma: correlation with hepatic metastasis and impact on prognosis. Cancer 2000; 88: 295–301. 10640960 10.1002/(sici)1097-0142(20000115)88:2<295::aid-cncr8>3.0.co;2-u

[bib27] Sawada M , Miyake S , Ohdama S , Matsubara O , Masuda S , Yakumaru K et al. Expression of tissue factor in non-small-cell lung cancers and its relationship to metastasis. Br J Cancer 1999; 79: 472–477. 10027315 10.1038/sj.bjc.6690073 PMC2362438

[bib28] Schaffner F , Versteeg HH , Schillert A , Yokota N , Petersen LC , Mueller BM et al. Cooperation of tissue factor cytoplasmic domain and PAR2 signaling in breast cancer development. Blood 2010; 116: 6106–6113. 20861457 10.1182/blood-2010-06-289314 PMC3031395

[bib29] Ruf W , Yokota N , Schaffner F . Tissue factor in cancer progression and angiogenesis. Thromb Res 2010; 125: S36–S38. 20434002 10.1016/S0049-3848(10)70010-4 PMC3827916

[bib30] Poon RT , Lau CP , Ho JW , Yu WC , Fan ST , Wong J . Tissue factor expression correlates with tumor angiogenesis and invasiveness in human hepatocellular carcinoma. Clin Cancer Res 2003; 9: 5339–5345. 14614019

[bib31] Rullier A , Senant N , Kisiel W , Bioulac-Sage P , Balabaud C , Le Bail B et al. Expression of protease-activated receptors and tissue factor in human liver. Virchows Arch 2006; 448: 46–51. 16193294 10.1007/s00428-005-0078-0

[bib32] Toomey JR , Kratzer KE , Lasky NM , Broze GJ Jr . Effect of tissue factor deficiency on mouse and tumor development. Proc Natl Acad Sci USA 1997; 94: 6922–6926. 9192667 10.1073/pnas.94.13.6922 PMC21260

[bib33] Bromberg ME , Sundaram R , Homer RJ , Garen A , Konigsberg WH . Role of tissue factor in metastasis: functions of the cytoplasmic and extracellular domains of the molecule. Thromb Haemost 1999; 82: 88–92. 10456459

[bib34] Palumbo JS , Talmage KE , Massari JV , La Jeunesse CM , Flick MJ , Kombrinck KW et al. Tumor cell-associated tissue factor and circulating hemostatic factors cooperate to increase metastatic potential through natural killer cell-dependent and-independent mechanisms. Blood 2007; 110: 133–141. 17371949 10.1182/blood-2007-01-065995 PMC1896107

[bib35] Liotta LA . Tumor invasion and metastases—role of the extracellular matrix: Rhoads Memorial Award lecture. Cancer Res 1986; 46: 1–7. 2998604

[bib36] Iaccarino I , Martins LM . Therapeutic targets in cancer cell metabolism and death. Cell Death Differ 2011; 18: 565–570. 21212794 10.1038/cdd.2010.174 PMC3131995

[bib37] Steeg PS . Perspective: the right trials. Nature 2012; 485: S58–S59. 22648501 10.1038/485S58a

[bib38] Block TM , Mehta AS , Fimmel CJ , Jordan R . Molecular viral oncology of hepatocellular carcinoma. Oncogene 2003; 22: 5093–5107. 12910247 10.1038/sj.onc.1206557

[bib39] Bosch FX , Ribes J , Diaz M , Cleries R . Primary liver cancer: worldwide incidence and trends. Gastroenterology 2004; 127: S5–S16. 15508102 10.1053/j.gastro.2004.09.011

[bib40] Zacharski LR , Henderson WG , Rickles FR , Forman WB , Cornell CJ Jr. , Forcier RJ et al. Effect of warfarin anticoagulation on survival in carcinoma of the lung, colon, head and neck, and prostate. Final report of VA Cooperative Study #75. Cancer 1984; 53: 2046–2052. 6322957 10.1002/1097-0142(19840515)53:10<2046::aid-cncr2820531007>3.0.co;2-f

[bib41] Gomez-Outes A , Suarez-Gea ML , Lecumberri R , Rocha E , Pozo-Hernandez C , Vargas-Castrillon E . New parenteral anticoagulants in development. Ther Adv Cardiovasc Dis 2011; 5: 33–59. 21045018 10.1177/1753944710387808

[bib42] Bruix J , Sherman M , Llovet JM , Beaugrand M , Lencioni R , Burroughs AK et al. Clinical management of hepatocellular carcinoma. Conclusions of the Barcelona-2000 EASL conference. European Association for the Study of the Liver. J Hepatol 2001; 35: 421–430. 11592607 10.1016/s0168-8278(01)00130-1

[bib43] Weidner N , Semple JP , Welch WR , Folkman J . Tumor angiogenesis and metastasis—correlation in invasive breast carcinoma. N Engl J Med 1991; 324: 1–8. 10.1056/NEJM199101033240101 1701519

