# Supplementary figures and images for: Factor VII promotes hepatocellular carcinoma progression through ERK-TSC signaling
Source: Cell Death Discov. 2015 Nov 30;1:15051–. doi: 10.1038/cddiscovery.2015.51 (PMC4993037; doi:10.1038/cddiscovery.2015.51)

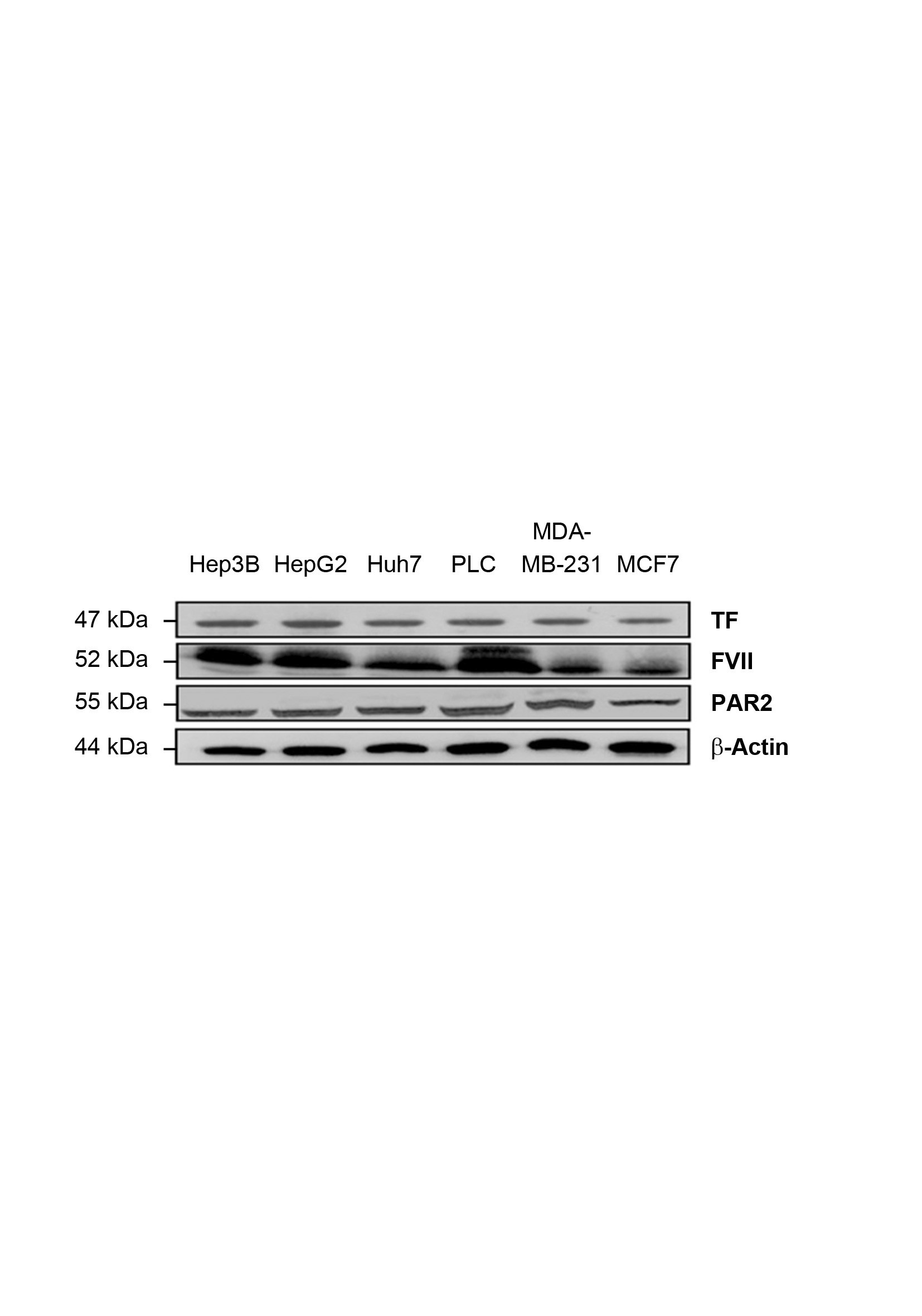

Supplement: Supplementary Figure 1 [file cddiscovery201551-s1.jpg]

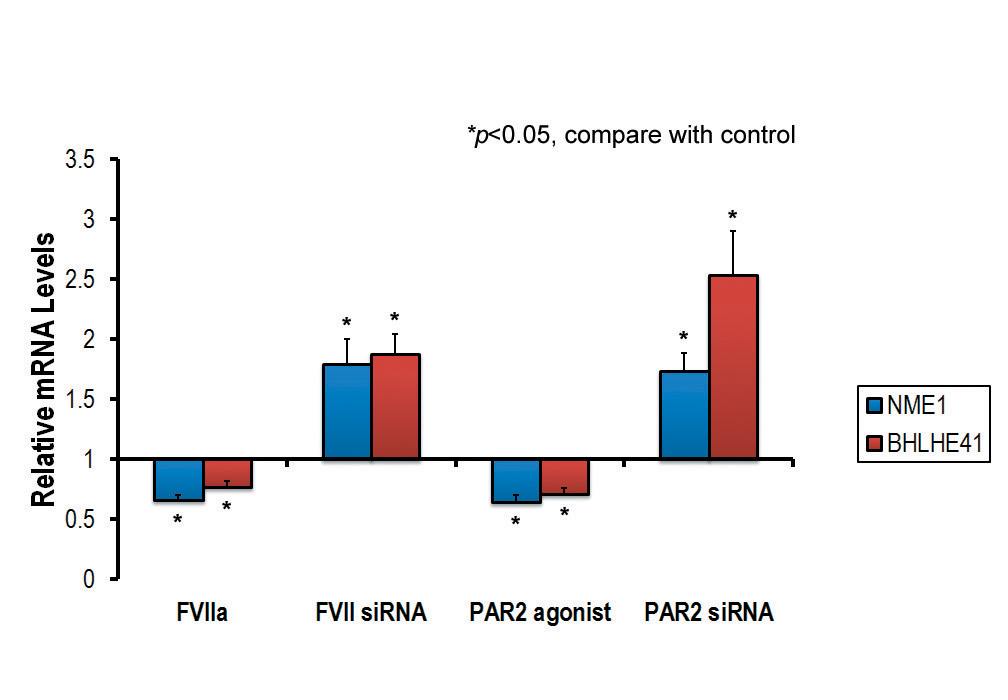

Supplement: Supplementary Figure 2 [file cddiscovery201551-s2.jpg]
